# Data on perceived excessive workload on faculty members׳ commitment

**DOI:** 10.1016/j.dib.2018.08.132

**Published:** 2018-08-31

**Authors:** Olumuyiwa A. Oludayo, Comfort O. Akanbi, Hezekiah O. Falola, Oluwafisayo A. Aluko

**Affiliations:** a Department of Business Management, Covenant University, Ota, Nigeria; b Department of Economics, Bowen University, Iwo, Nigeria

**Keywords:** Workload, Organization, Commitment, Faculty members

## Abstract

For this article, the survey data on the effect of perceived excessive workload on faculty members’ commitment was presented. This data was gathered from an academic environment using the full time faculty members of Covenant University. The descriptive research design method was employed. The initial sample size used for the analysis was 228 faculty members but only 189 copies of questionnaire were returned. Statistical package for social sciences (SPSS) was used for the coding of the data. The validity and reliability of the research instrument were carried out using Cronbach Alpha. Descriptive analysis was used for the presentation of the data. This data is made publicly available to assist further study in the area of workload and employees commitment.

**Specifications Table**

**Table:** 

Subject area	*Management*
More specific subject area	*Human Resource Management*
Type of data	*Charts, Table*
How data was acquired	*The data was gotten from the distribution of copies of questionnaire to faculty members of Covenant University*
Data format	*Raw, analyzed, descriptive research design*
Experimental factors	*Sample consisted of academic staff of Covenant University. The researcher-made questionnaire which contained data on excessive work-load and faculty commitment were completed.*
Experimental features	*Descriptive data are presented using tables.*
Data source location	*Covenant University, Canaan Land, Ota, Nigeria (Latitude 6.6718*^*o*^*N, Longitude 3.1581*^*o*^*E)*
Data accessibility	*In order to encourage evidence-based research in work-life balance in organizations, detailed dataset are made publicly available.*
Related research article	*Nil*

**Value of the data**

• The data provided in this article shows the basic criteria for measuring excessive workload in organizations and could be adopted for further research work.
• This dataset indicates that there is a link between excessive workload and commitment of employees, thus, its availability will foster the need to emphasize on the subject matter.
• Improved research work on this survey data would enhance faculty members’ commitment by increased morale, motivation and reduced absenteeism.
• Additional investigation of the survey data by the universal professional research bodies will increase the understanding of the effect of work overload on faculty commitment across countries.
• Data shared in this data article will open up doors for new research collaborations.

## 1 Data

**Fig. 1:**
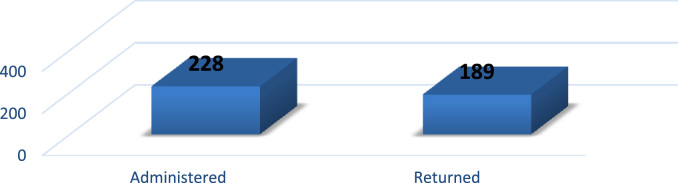
Rate of response to survey questionnaire.

**Fig. 2:**
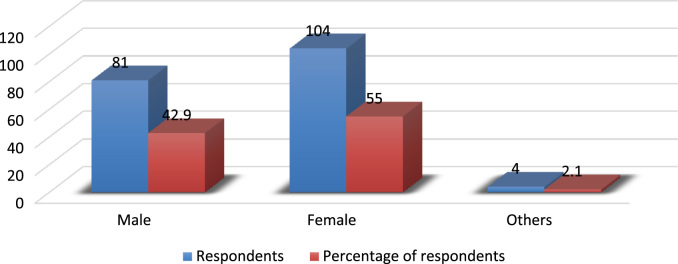
Demographic analysis by gender. Source: Field survey, 2018

**Fig. 3:**
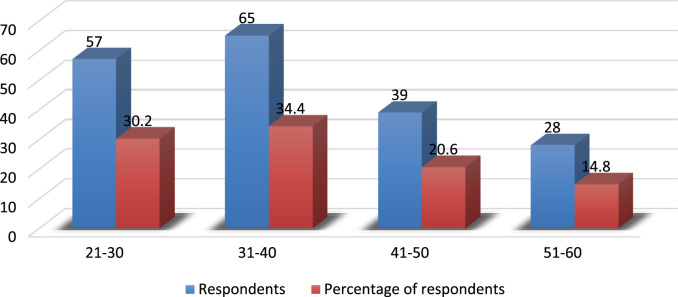
Demographic analysis by age. Source: Field survey, 2018

**Fig. 4:**
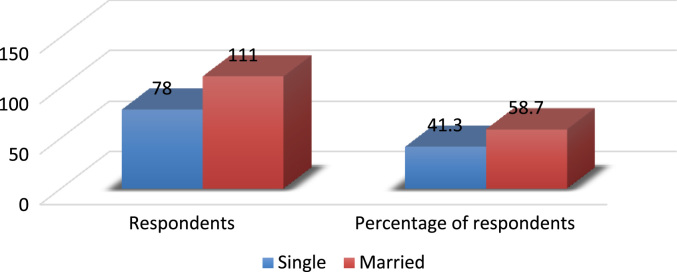
Demographic analysis by marital status. Source: Field survey, 2018

**Fig. 5:**
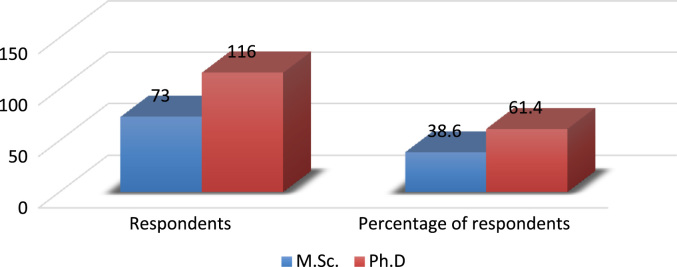
Demographic analysis by educational attainment. Source: Field survey, 2018

**Fig. 6:**
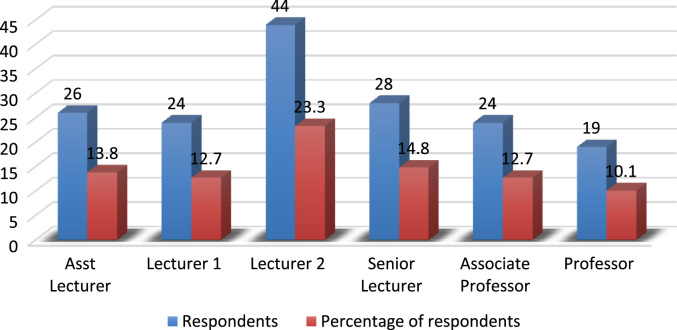
Demographic analysis by academic position. Source: Field survey, 2018

The evolving competition in the university system has placed pressure on employees’ commitment particularly in a recessed economy where organisations are reducing the overhead cost by reducing the workforce and increasing the workload of the surviving employees. Active engagement of faculty members in research, teaching and community impact are *sine qua non* for a university׳s survival [3], [4], [5], [6], [7]. In order to investigate the effect of perceived excessive workload on faculty members’ commitment, a survey was carried out using Covenant University. The Data presented in this study is quantitative and descriptive in nature. For the coding of data collected, Statistical Package for Social Sciences (SPSS) was used. Fig. 1 shows the response rate of questionnaire administered while the demographic characteristics of the respondents are depicted in Fig. 2, Fig. 3, Fig. 4, Fig. 5, Fig. 6. In a related development, descriptive analysis of responses on the effect of perceived excessive workload on faculty members’ commitment is depicted in Table 1. Similarly, perceived excessive workload on faculty members’ commitment was tested using five Likert scale as suggested by [1], [2]. This helps in determining the faculty members’ view and the level at which they agree to each item in the questionnaire. The Likert scale is represented as: Strongly Agree (SA), Agree (A), Neutral (N), Disagree (D) and Strongly Disagree (SD) with specific value of 5,4,3,2,1 in that order. The data will be very useful for university management, National Universities Commission, Ministry of Education and human resource policy makers in addressing issues that affect excessive workload and faculty commitment.

Number 1 shows the response to the statement “Effective work life balance initiative helps to reduce excessive workload”. 5 (2.6%) respondents were neutral about the statement. 85 (45.0%) respondents agreed that effective work life balance initiative helps to reduce excessive workload while 99 (52.4%) of the respondents strongly agreed that effective work life balance initiative helps to reduce excessive workload.
Number 2 presents the responses to the research question are the work life balance initiatives in your organization suitable? Only 1 respondent strongly disagreed with the question representing 0.5%, 3 respondents disagreed representing 1.6%, 14 (7.4%) respondents were neutral about the question. 125 (66.1%) respondents agreed while 46 (24.3%) respondents strongly agreed to the question.
Number 3 presents the summary of the data on the responses to the research statement “I often feel that the work life balance initiatives are not effective.”18 (9.5%) respondents strongly disagreed with the statement, 28 (14.8%) disagreed with the statement, 22 (11.6%) respondents were neutral on the statement, 64 (33.9%) respondents agreed with the statement and 57 (30.2%) respondents strongly agreed to the statement.
Number 4 provides responses to the statement “I sometimes stay back to finish my work for the day”. 5(2.6%) of the respondents strongly disagreed, 4 (2.1%) of the respondents disagreed while 8 (4.2%) respondents were neutral. 94 (49.7%) of the respondents agreed and 78 (41.3%) strongly agreed that they often stayed back to finish the work for the day.
Number 5 shows responses to the statement on I always make sure I go early to work or other related activities. 10 (5.3%) respondents strongly disagreed with the statement, 1 (0.5%) respondent disagreed, 9 (4.8%) respondents were neutral on the statement, 139 (73.5%) respondents agreed and 30 (15.9%) respondents strongly agreed.
Number 6 Presents responses to whether the respondents were interested in everything that goes on in the organization. 9 (4.8%) of the respondents strongly disagreed, 18 (9.5%) of the respondents disagreed while 19 (10.1%) of the respondents were neutral as regards the notion. 76 (40.2%) agreed and 67 (35.4%) strongly agreed that they were interested in everything that goes on in the organization.

**Table 1:** Descriptive analysis of responses on perceived excessive workload on faculty members’ commitment.

**S/N**	**View**	**SA 5%**	**A 4%**	**N 3%**	**D 2%**	**SD 1%**
1	Effective work life balance initiative helps to reduce excessive workload	99 (52.4)	85 (45.0)	5 (2.6)	0	0
2	Work life balance initiatives in my institution is suitable	46 (24.3)	125 (66.1)	14 (7.4)	3 (1.6)	1 (0.5)
3	Oftentimes work life balance initiatives of my institution are not effective	57 (30.2)	64 (33.9)	22 (11.6)	28 (14.8)	18 (9.5)
4	Sometimes I stay back in the office to finish work for the day.	78 (41.3)	94 (49.7)	8 (4.2)	4 (2.1)	5 (2.6)
5	Always go early to work and other related activities	30 (15.9)	139 (73.5)	9 (4.8)	1 (0.5)	10 (5.3)
6	I am interested in everything that goes on in my institution	67 (35.4)	76 (40.2)	19 (10.1)	18 (9.5)	9 (4.8)

## 2 Experimental design, materials, and methods

Data were collected from the sample of two hundred and twenty eight (228) faculty members of Covenant University with the aid of structured questionnaire designed by the researcher based on the similar studies. Stratified and simple random sampling techniques were used. Copies of questionnaire were administered directly to all categories of faculty members ranging from Assistant Lecture to Professor. What informed the choice of the selected Covenant University was because of the fact that Covenant University has been best private university in Nigeria for over four years consecutively. Faculty members were also purposefully selected because of the university drive for quality research outputs and excellent teaching delivery toward achieving their vision of becoming one of the best universities in the world. Two hundred and twenty eight (228) copies of questionnaire were administered; one hundred and eighty nine (189) copies of the questionnaire were retrieved from the field giving the response rate of (82.9%). Meanwhile, the researchers also sought for the permission of the management of the selected institution before the questionnaire were administered to them. In addition, every participant was adequately informed about the objective of the study. In addition, all respondents were given opportunity to stay anonymous and their responses were treated with upmost confidentiality.
